# Evaluation of Subclinical Cancer Therapy-Related Cardiac Dysfunction in Patients Undergoing Hematopoietic Stem Cell Transplantation: An Echocardiography Study

**DOI:** 10.3390/cancers16223808

**Published:** 2024-11-12

**Authors:** Audrone Vaitiekiene, Migle Kulboke, Monika Bieseviciene, Austeja Kaunaite, Auste Markeviciute, Agne Bartnykaite, Benas Kireilis, Diana Rinkuniene, Antanas Jankauskas, Ignas Gaidamavicius, Rolandas Gerbutavicius, Domas Vaitiekus, Gintare Sakalyte, Jolanta Justina Vaskelyte

**Affiliations:** 1 Department of Cardiology, Medical Academy, Lithuanian University of Health Sciences, 44307 Kaunas, Lithuania; 2 Department of Oncology and Hematology, Medical Academy, Lithuanian University of Health Sciences, 44307 Kaunas, Lithuania; 3 Oncology Institute, Lithuanian University of Health Sciences, 50161 Kaunas, Lithuania; 4 Medical Academy, Lithuanian University of Health Sciences, 44307 Kaunas, Lithuania; 5 Oncology Research Laboratory, Oncology Institute, Lithuanian University of Health Sciences, 50161 Kaunas, Lithuania; 6 Department of Radiology, Medical Academy, Lithuanian University of Health Sciences, 44307 Kaunas, Lithuania; 7 Institute of Cardiology, Lithuanian University of Health Sciences, 47181 Kaunas, Lithuania

**Keywords:** cancer therapy-related cardiac dysfunction, cardiotoxicity, hematopoietic stem cell transplantation, cardiovascular complications, echocardiography

## Abstract

**Background:** Hematopoietic stem cell transplantation (HSCT) is a potentially curative procedure that is used in various hematological malignancies. However, among an increasing number of HSCT, the amount of cancer therapy-related cardiac dysfunction (CTRCD) is increasing as well. This study aimed to determine the prevalence of subclinical CTRCD in HSCT patients 12 months after HSCT and to assess the impact of clinical factors on the development of CTRCD. **Material and Methods**: We included 55 patients who underwent autologous or allogeneic HSCT. The patients were assessed using an echocardiography method before and 12 months after a HSCT procedure. **Results:** Our study revealed that during the 12-month follow-up period, asymptomatic CTRCD was observed in 15 patients (27.3%), 6 experienced moderate CTRCD, and 9 experienced mild CTRCD. Patients with previous use of anthracyclines tended to have CTRCD more often: nine patients (60%) in the CTRCD group and nine patients (22.5%) in non-CTRCD group. Patients who received the BEAM regimen for conditioning also experienced CTRCD more often: five patients (33.3%) in CTRCD group vs. two patients (5%) in the non-CTRCD group. **Conclusions**: Our study showed that asymptomatic CTRCD was found in 27.3% of the patients 12 months after HSCT. The BEAM chemotherapy conditioning protocol following prior anthracycline use were identified as factors contributing to the development of CTRCD.

## Simple Summary

Increasing number of hematopoietic stem cell transplantation (HSCT) and prolonged survival rates of these patients lead to the increase of cancer therapy-related dysfunction (CRTCD) as well. The study aimed to determine the prevalence of subclinical CTRCD in HSCT patients 12 months after HSCT and to assess the impact of clinical factors on the development of CTRCD. The study involved 55 patients who had undergone either autologous or allogeneic HSCT. Echocardiography scans were performed to check the patients’ cardiac function before the transplant and again 12 months after. The study revealed that asymptomatic CTRCD was found in 27.3% of the patients 12 months after HSCT. The BEAM chemotherapy conditioning protocol following prior anthracycline use were identified as factors contributing to the development of CTRCD, therefore, these patients should undergo closer follow-up and start cardioprotective treatment when indicated.

## 1. Introduction

Hematopoietic stem cell transplantation (HSCT) is a potentially curative therapy frequently used in patients with hematological malignancies (HM), nonmalignant bone marrow disorders, and certain solid tumors [1]. The HSCT procedure involves a process called conditioning, which involves a bone marrow ablation. This is achieved either through the administration of a high dose of chemotherapy or total-body irradiation. After conditioning, an intravenous injection of hematopoietic stem cells is administered, which can be autologous or allogeneic [2]. In autologous HSCT, the stem cells are derived from the patient, whereas in allogeneic HSCT, they are obtained from a donor [3].

With the increasing indications for a HSCT procedure, the number of treatment-related complications, such as graft-versus-host disease in allogeneic transplantations or cardiovascular complications (CVCs), are increasing as well. According to the Worldwide Network of Blood and Marrow Transplantation (WBMT), the number of patients receiving HSCT has been rising for the past decade at a rate of more than 7% per year over the past decade, with an average of 90,000 HSCT procedures performed annually [4]. Although the survival rates and clinical outcomes for patients post-HSCT have improved over the past few decades, the risk of developing at least one procedure-related long-term complication remains high among survivors [1]. While CVC accounts for less than 10% of the overall complications associated with HSCT, they can lead to higher mortality rates and significantly reduced quality of life in long-term survivors [5,6].

Cardiac adverse events are associated with various components of HSCT, one of which is bone marrow ablation therapy, including total body irradiation combined with a multidrug conditioning regimen [7]. The majority of the drugs used for mobilization or conditioning, which include cyclophosphamide, cytarabine, or carmustine, are associated with significant toxicity. In addition, the effects of dimethyl sulfoxide, which is used to preserve stem cells, are also thought to contribute to cardiac events [8].

There has been an increasing number of studies that recommend reducing cardiac-related morbidity and CVC by identifying at-risk subpopulations. However, there is no standardized approach for evaluating cardiac risk in transplant candidates [1].

This study aimed to determine the prevalence of subclinical cancer therapy-related cardiac dysfunction (CTRCD) in HSCT patients 12 months after HSCT and to assess the impact of clinical factors on the development of CTRCD. Two-dimensional echocardiography imaging and CTRCD diagnostic criteria in the guidelines of the European Society of Cardiology (ESC) developed in collaboration with EHA/ESTRO/IC-OS (2022) were used to evaluate possible cardiotoxicity following a HSCT procedure [9].

## 2. Materials and Methods

The study was performed prospectively at the Hospital of the Lithuanian University of Health Sciences Kaunas Clinics. The study took place from October 2021 to February 2024 and involved 55 patients who underwent autologous or allogeneic HSCT at the Department of Oncology and Hematology. Kaunas regional Bioethics Committee permission was obtained for this prospective research (No. BE-2-96). The research was carried out in accordance with the Helsinki Declaration. All the patients gave their informed consent to take part in this study.

The inclusion criteria were as follows:

- Written informed consent to participate in the study;
- Patients over the age of 18 at the time of HSCT procedure.

The exclusion criteria were as follows:

- Previously performed HSCT;
- Refusal to participate in the study at any point.

During the study, after assessing the inclusion and exclusion criteria and signing the informed consent forms, the patients were surveyed and asked to complete a questionnaire regarding cardiovascular (CV) disease risk factors (smoking, dyslipidemia, diabetes), current or past history of CV diseases (hypertension, early coronary heart disease (CHD), and CV medication used. Hypertension was assessed according to the European Society of Cardiology and European Society of Hypertension guidelines published in 2018 [10]. Hypertension was diagnosed in cases where systolic blood pressure was ≥140 mmHg and/or diastolic blood pressure was ≥90 mmHg. Early CHD was defined as a cardiovascular event (stroke, myocardial infarction, interventional procedure, or cardiac revascularization) or the death of a first-degree relative due to a cardiovascular event (men < 55 years, women < 60 years).

The patients underwent HSCT for various reasons. The distribution of the diseases and types of transplantation performed is described in the results section.

During autologous HSCT, hematopoietic stem cells were collected following chemotherapy and the administration of granulocyte colony-stimulating factors (G-CSF) (Filgrastim 10 µg/kg/d). Multiple myeloma patients had cyclophosphamide 3000 mg/m^2^; the mantle cell lymphoma patients had rituximab 375 mg/m^2^ and cytarabine 4 g/m^2^; the Hodgkin’s lymphoma patients had cisplatin 100 mg/m^2^ and cytarabine 4 g/m^2^; the primary central nervous system (PCNS) diffuse large B cell lymphoma patients had cytarabine and thiotepa at different doses and rituximab 375 mg/m2; the NK/T-cell lymphoma patient had CHOEP (cyclophosphamide, doxorubicin, etoposide, vincristine, and prednisone) scheme; and the Ewing sarcoma patient followed an IE scheme (ifosfamide with etoposide). Apheresis procedures were performed on the patients undergoing autologous HSCT. Stem cells were collected from the peripheral blood using the Fresenius Kabi COM.TEC apheresis system, and the cells were cryopreserved in 10% DMSO and an autologous plasma solution. The cryopreservation process was carried out in a controlled-rate freezer with liquid nitrogen (Consarctic equipment).

Conditioning chemotherapy was administered according to the disease: for multiple myeloma, melphalan 200 mg/m^2^ was used, and for lymphomas, the BEAM protocol (carmustine, etoposide, cytarabine, and melphalan) was applied. For PCNS, diffuse large B-cell lymphoma, thiotepa and BCNU (carmustine) were administered. For the patients undergoing allogeneic HSCT, conditioning involved reduced-intensity fludarabine and busulfan.

**Figure 1:**
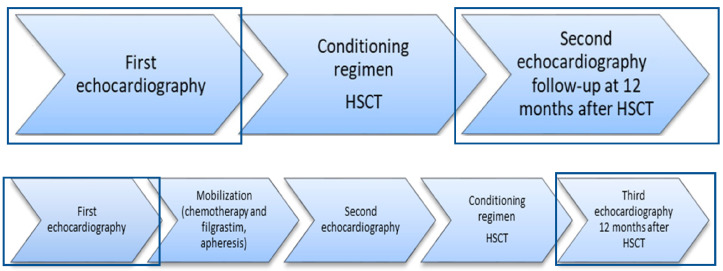
Echocardiography workflow of the study. The first diagram shows allogeneic HSCT process, and the second diagram shows autologous HSCT process. HSCT—hematopoietic stem cell transplantation.

Echocardiography was performed three times for the patients undergoing autologous HSCT: first, before the mobilization procedure, to assess the cardiac status before deciding to include the patient in the transplantation process; second, before the transplantation (the conditioning regimen) procedure; and third, during a follow-up, 12 ± 1 months after HSCT. Echocardiography was performed two times for patients undergoing allogenic HSCT: first, to assess cardiac status before deciding to include the patient in the transplantation process, second, during a follow-up, 12 ± 1 months after HSCT. In this study, we aimed to determine the effect of autologous and allogeneic HSCT on the heart; therefore, the echocardiographic parameters of the patients before the decision to include them in the transplantation process and 12 ± 1 months after the HSCT were analyzed. Echocardiography workflow is presented in Figure 1.

Echocardiography was performed and evaluated by one experienced cardiologist using a EPIQ 7 (Phillips Ultrasound Inc., Bothell, WA, USA) ultrasound machine. The routine echocardiographic parameters (quantification of cardiac chamber size and function) and the global longitudinal strain were measured.

The quantification of the cardiac chamber size and function were measured according to the American Society of Echocardiography and the European Association of Cardiovascular Imaging recommendations for Cardiac Chamber Quantification [11].

The following measurements were obtained from the parasternal long-axis view—the left ventricular end-diastolic diameter (LVEDD), mm, and its index calculated according to the body surface area (BSA) (LVEDDi), mm/m^2^. The left ventricular (LV) mass (LVM) was calculated using the Cube formula (0.8 × (1.04 × (IVS+LVID+PWT) ^3^ − LVID ^3^)+ 0.6 g (IVS—interventricular septum; LVID—left ventricle internal diameter; PWT—inferolateral wall thickness)), and the relative wall thickness (RWT) was calculated using the following formula (2 × PWT)/LVID. An increase in the LV mass was categorized as either concentric (RWT > 0.42) or eccentric (RWT ≤ 0.42) hypertrophy. The normal LV mass with increased RWT > 0.42 was categorized as concentric remodeling. The left atrium (LA) diameter, mm, at end-systole was also measured.

In the apical views, the left ventricle ejection fraction (LVEF) was calculated, and the global LVEF was assessed by calculating the difference between the left ventricular (LV) end-diastolic and end-systolic volumes, divided by the LV end-diastolic volume. The LA volume, ml, was calculated from four- and two-chamber views using the disk summation algorithm, and the LA volume index was calculated based on the BSA, ml/m^2^.

The right ventricular (RV) longitudinal systolic function was assessed by measuring the RV free wall peak systolic velocity using a tissue doppler (S′, cm/s). A tissue doppler was also used to assess the early LV diastolic filling velocities (e′) at the lateral LV wall and the septum (cm/s) and the E/e′ ratio (e′—the average of the lateral LV wall and septum). Global longitudinal strain (GLS) of the LV was assessed using speckle-tracking method, calculated by analyzing four-, three-, and two-chamber apical views. The GLS was calculated from one single, high-quality cardiac cycle image. After calculating the longitudinal strains of all the LV walls, the GLS was determined as an arithmetic mean of all the myocardial segments. Echocardiographic measurements were collected before and after allogeneic and autologous HSCT, and a statistical data analysis was performed.

**Figure 2:**
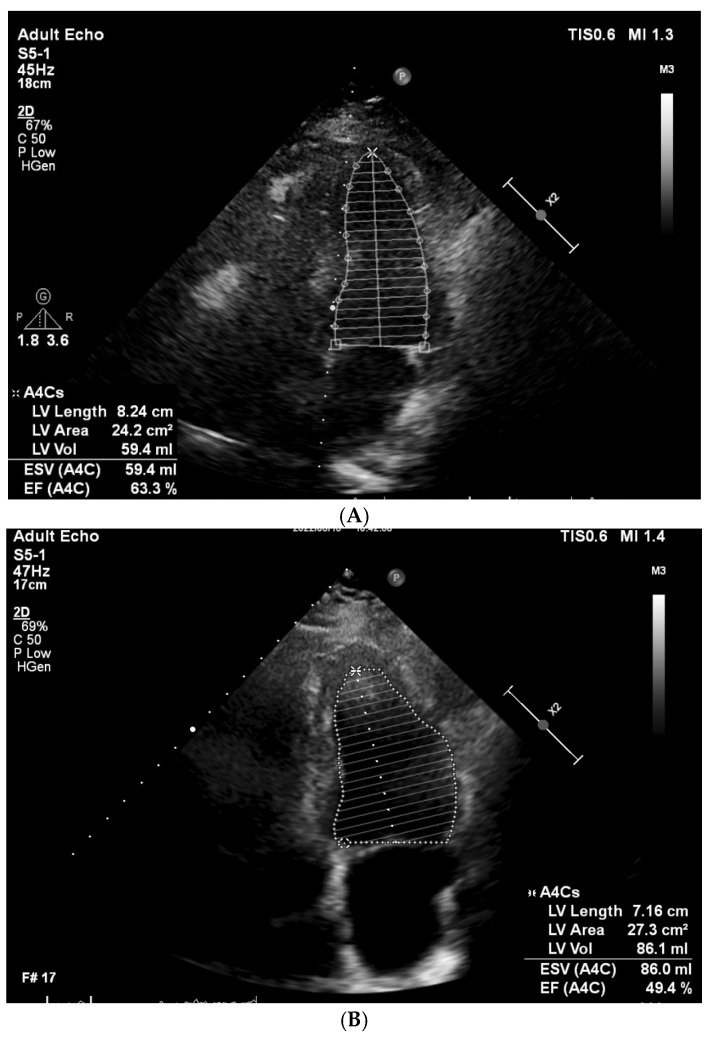
Change in LVEF from 63.3% to 49.4%, calculated using the modified biplane Simpson’s method. (**A**) baseline echocardiography; (**B**) follow-up echocardiography 12 months after HSCT. LVEF: left ventricular ejection fraction, HSCT: hematopoietic stem cell transplantation.

**Figure 3:**
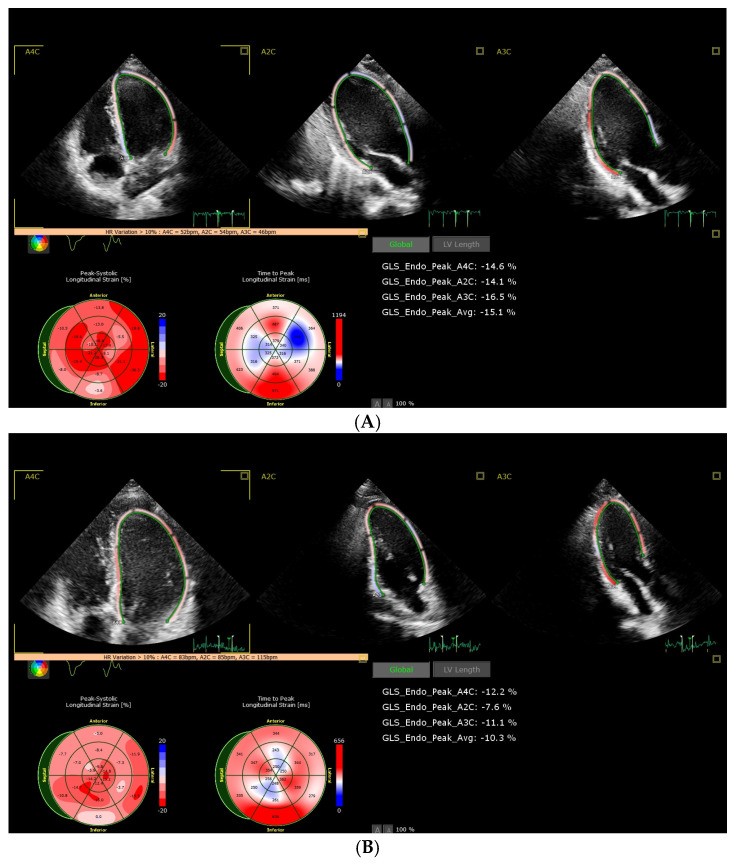
Change in GLS from −15.1% to −10.3%. (**A**) baseline echocardiography; (**B**) control echocardiography 12 months after HSCT. GLS: global longitudinal strain, HSCT: hematopoietic stem cell transplantation.

According to the European Society of Cardiology’s (ESC) cardio-oncology guidelines’ diagnostic criteria, developed in collaboration with EHA/ESTRO/IC-OS and published in 2022 [9], the patients were divided into two groups, with subclinical CTRCD or without CTRCD, based on the change in the following echocardiographic parameters: LVEF and GLS. In our study, we aimed to assess the prevalence of CTRCD and the risk factors for the development of CTRCD. The CTRCD diagnostic criteria and patient classification are presented in Table 1. The examples of the baseline and follow-up cardiography studies are presented in Figure 2 and Figure 3.

A statistical analysis was performed using the IBM SPSS Statistics 29.0 software package (IBM Corp., Armonk, NY, USA). The qualitative variables were described by absolute numbers (n) with percentage expression (%); the quantitative variables meeting normality conditions were presented as means with standard deviations, and those not meeting normality conditions were presented as medians with minimum and maximum values. A paired *t*-test or Wilcoxon test were used to compare the data between two groups of observed measurements. The relationship between the development of cardiotoxicity and the clinical features of the patients was assessed using Pearson’s chi-square or Fisher’s Exact test. The binary logistic regression analysis was performed with univariate and multivariate models to estimate the odds ratio (OR) with a 95% confidence interval (CI). The bootstrap method was applied to the multivariate logistic regression, with bias-corrected and accelerated (BCa) bootstrap confidence intervals (the results were based on 1000 bootstrap samples). A statistically significant change was defined as *p* < 0.05.

## 3. Results

### 3.1. Clinical and Demographic Characteristics

The data of 55 patients were analyzed. There were 30 men (54.5%) and 25 women (45.5%). The median age was 61 years, ranging from 18 to 74. A total of 48 patients (87.3%) underwent autologous HSCT and 7 patients (12.7%) underwent allogeneic HSCT. The main characteristics of the patients and the main diseases are listed in Table 2.

### 3.2. Analysis of CTRCD and Factors Influencing and Prognosing CTRCD

Three patients (5.5%) experienced clinically significant cardiovascular symptoms during the 12 months period after HSCT, all of which were supraventricular arrhythmia: two experienced supraventricular tachycardia and one experienced atrial fibrillation.

According to the ESC guidelines on cardio-oncology, the 2022 definition, and the echocardiographic parameters, 15 patients (27.3%) had asymptomatic CTRCD at the 12-month follow-up [9]. Six of the patients had moderate (either a new LVEF reduction by ≥10% to an LVEF of 40–49% or a new LVEF reduction by <10% to an LVEF of 40–49% and a new relative decline in GLS by >15% from the baseline), and nine patients had mild CTRCD (an LVEF ≥ 50% and a new relative decline in GLS by >15% from the baseline). Detailed information about each patient with CTRCD is presented in the Supplementary Table S1. The characteristics of the patients and the differences among the CTRCD and non-CTRCD groups are presented in Table 3.

The patients’ age, sex, main disease, type of HSCT, CV risk factors, and CV medication did not differ statistically significantly among the two groups. The patients with previous use of anthracyclines tended to have CTRCD more often: nine patients (60%) in the CTRCD group and nine patients (22.5%) in the non-CTRCD group. The difference was statistically significant (*p* = 0.021). There was also a difference in the conditioning regimens. The patients who received BEAM regimen for conditioning had CTRCD more often: five patients (33.3%) in the CTRCD group vs. two patients (5%) in the non-CTRCD group. The difference was statistically significant (*p* = 0.013).

Various factors, possibly influencing the development of CTRCD, including sex, the main disease, the type of HSCT, previous CV diseases, CV risk factors, the previous use of anthracyclines, and different conditioning regimens, were taken into account in a univariate logistic regression analysis. We found that the previous use of anthracyclines (OR 5.167, 95% CI 1.448–18.433, *p* = 0.011), and BEAM used for a conditioning regimen (OR 9.500, 95% CI 1.599–56.426, *p* = 0.013) were significant factors in the development of CTRCD (Table 4).

In order to find out whether the previous use of anthracyclines and BEAM conditioning regimen have impact on the development of CTRCD independently of different cardiovascular risk factors, we performed a multivariate logistic regression analysis. We determined that the statistically significant results from the univariate logistic regression analysis lost significance. This could have happened because the BEAM conditioning regimen and the previous use of anthracyclines could be two dependent variables—all the patients who received the BEAM conditioning regimen have previously had anthracycline-based chemotherapy (Supplementary Table S1). However, the bootstrap method (on 1000 bootstrap samples) in the multivariate logistic regression analysis shows that the effect of the BEAM for conditioning could be a potentially independent factor in the increased risk of CTRCD (*p* = 0.039, Table 5), but further investigation with a bigger sample is needed.

The impact of the BEAM conditioning regimen and the previous use of anthracyclines on the development of CTRCD independent of different cardiovascular risk factors was analyzed, performing a multivariate logistic regression analysis separately. We determined that the use of BEAM for conditioning had a significantly higher risk for CTRCD independent of cardiovascular risk factors (OR 14.910, 95% CI 1.764–126.038, *p* = 0.013, Table 6). The bootstrap method in the multivariate logistic regression analysis confirmed the effect of the BEAM on the increased risk of CTRCD (*p* = 0.006). Similar results were noticed with previous anthracycline use—the risk of CTRCD development was significantly higher (OR 6.996, 95% CI 1.530–31.997, *p* = 0.012, Table 7). The bootstrap method confirmed the results of the multivariate logistic regression analysis (*p* = 0.009).

## 4. Discussion

In our study, we aimed to assess the incidence of CTRCD after HSCT and identify factors that may influence and help to predict the development of CTRCD.

Our study revealed that during the 12-month follow-up period, asymptomatic CTRCD was observed in 15 patients (27.3%); 6 experienced moderate CTRCD and 9 experienced mild CTRCD according to the criteria of the ESC guidelines on cardio-oncology in 2022. Other studies defined CTRCD according to different criteria. Moriyama and colleagues conducted a retrospective study and assessed the early onset of CTRCD after allogeneic HSCT. They reviewed the records of 136 patients and defined LV systolic dysfunction as a decrease in LVEF by ≥10% or an LVEF ≤53% over a period of 100 days after HSCT. The incidence of CTRCD in the study was 17%. They also observed that patients presenting with early CTRCD had higher mortality from a primary disease when compared to those without early CTRCD [12]. There are several more studies where late CTRCD is analyzed post-HSCT. One study analyzed 274 patients after autologous HSCT, defining LV systolic dysfunction as an LVEF <50%. The results were compared with a control group that matched the age and gender of the patients. CTRCD was found in 15.7% of patients and 5.1% of them were asymptomatic [13]. Although different methods were used in these studies, their results indicate that the incidence of LV systolic dysfunction increases several years after HSCT.

Concerning the factors possibly influencing the development of CTRCD, our study did not reveal one significant factor. A univariate logistic regression analysis showed that the BEAM conditioning regimen and previous use of anthracyclines could be the factors influencing the development of CTRCD. However, the statistical significance in the multivariate logistic regression was lost. This could have probably happened because all the patients who had BEAM conditioning regimen were lymphoma patients and all of them had previously received anthracycline-based chemotherapy. Therefore, in our study these two factors were not independent. Nevertheless, a Bootstrap analysis revealed that the BEAM conditioning regimen potentially could be an independent factor, but the further analysis of a bigger sample is needed.

The BEAM conditioning regimen protocol consists of carmustine, etoposide, cytarabine, and melphalan. It is known that cytarabine and melphalan increase the incidence of heart failure [14,15]. Etoposide affects topoisomerase 2 inhibiting mitochondrial biogenesis. The development of CTRCD after the BEAM conditioning regimen in HSCT patients involves many mechanisms, such as direct damage to cardiac tissue, oxidative stress, vascular injury, inflammation, and cardiac fibrosis. It can lead to the increased production of reactive oxygen species (ROS) and interfere with the antioxidant defense mechanisms of the cardiomyocytes. This results in the decreased ability of the mitochondria to produce energy, as they are particularly sensitive to oxidative damage, leading to mitochondria-induced cell apoptosis. Another mechanism, related to CTRCD development, is the chemotherapy-induced activation of inflammatory pathways in the myocardium, which results in the release of pro-inflammatory cytokines and the infiltration of myocardium immune cells [16]. The resulting myocardial fibrosis increases myocardial stiffness and may lead to impaired diastolic function, contributing to systolic dysfunction and progressive heart failure. Over time, exposure to BEAM therapy may result in the loss of functional cardiomyocytes and increased ventricular dysfunction.

Anthracycline-induced cardiovascular complications are already well-analyzed and characterized, and their toxicities mostly directly correlate to their cumulative dose [17]. Other studies have also shown similar results regarding the development of CTRCD related to anthracycline use before HSCT [18,19,20]. Various studies indicate that CTRCD can occur even in patients without pre-existing cardiovascular risk factors [21]. The risk of developing CTRCD is particularly increased in the patient groups that receive high doses of anthracycline therapy or those with already-existing cardiovascular conditions [22,23]. The pathophysiology of CTRCD induced by anthracyclines involves several mechanisms. Firstly, anthracyclines may cause direct damage to cardiomyocytes, leading to left ventricular dysfunction. The main mechanism of this is through the inhibition of topoisomerase 2β. resulting in the activation of cell death pathways and the inhibition of mitochondrial biogenesis. This injury is often observed in patient groups receiving larger doses of chemotherapeutic agent, where higher cumulative doses result in greater risks of heart failure. Second, anthracyclines also induce oxidative stress and inflammation, which contributes to cell apoptosis and subsequently causes cardiac dysfunction. Recently published studies also suggest that anthracycline therapy may contribute to the aging processes of cardiac tissue, leading to vulnerability in heart disease later in life [24,25,26,27,28]. As the main mechanism of anthracycline-induced cardiotoxicity is thought to be the inhibition of topoisomerase 2β, etoposide from the BEAM conditioning regimen also has impact on topoisomerase 2. This could also be the reason why BEAM chemotherapy has further impact on the development of CTRCD.

Patients who develop early-onset CTRCD after HSCT also have worse overall survival rates compared to those without developed cardiac dysfunction. This emphasizes the need for early detection and intervention strategies to improve the outcomes in such patient populations [29,30]. Preventive strategies may include the use of cardioprotective agents during anthracycline therapy, although their efficacy varies. Continuous research is necessary to optimize treatment regimens that minimize cardiotoxic effects while maintaining cancer treatment efficacy [31].

According to the guidelines of cardio-oncology published by the European Society of Cardiology, the type of HSCT (with a higher risk after allogeneic HSCT), numerous uncontrolled cardiovascular risk factors, pre-existing cardiovascular diseases, and the direct cardiotoxic effects of anti-cancer medications all have a negative effect on the development of CTRCD [9]. Although, in our study, there were no statistically significant results regarding the type of HSCT; as only seven patients received allogeneic HSCT, there was a tendency of cardiotoxicity—three out of seven of the allogeneic HSCT patients developed CTRCD. We also did not obtain a statistically significant difference in the development of CTRCD regarding the cardiovascular risk factors. There are numerous studies analyzing the impact of cardiovascular risk factors in patients with different chemotherapy regimens. The impact of arterial hypertension on the development of cardiotoxicity in analyzed most widely; studies have noticed the significant impact [32,33]. Due to a relatively small sample in our study, the impact of the cardiovascular risk factors on the development of CTRCD could have been underscored. Therefore, further studies should be carried out and clinicians should aim to modify cardiovascular risk factors as much as possible.

### Limitations

The main limitation of this study is its relatively small sample size. Bootstrap analysis shows promising results regarding the BEAM conditioning regimen as an independent factor contributing to the development of CTRCD. Therefore, further investigation with a larger number of patients is needed to draw significant conclusions. Also, the impact of cardiovascular risk factors on the development of CTRCD could have been underestimated.

Moreover, due to a small number of patients undergoing allogeneic HSCT in our study, its effects on the development of CTRCD could have been underscored—we did not obtain statistically significant results.

## 5. Conclusions

Asymptomatic CTRCD, 12 months after HSCT, was found in 27.3% of the patients. The BEAM chemotherapy conditioning protocol following prior anthracycline use was identified as a factor contributing to the development of CTRCD.

## Figures and Tables

**Table 1:** Definition criteria of CTRCD and distribution of patients.

Grade	Criteria	Number of Patients
Severe	New LVEF reduction to <40%	0
Moderate	New LVEF reduction by ≥10% to LVEF of 40–49%	3
	New LVEF reduction by <10% to LVEF of 40–49% AND new relative decline in GLS by >15% from baseline	3
Mild	LVEF ≥50% AND new relative GLS reduction >15% from baseline	9

LVEF—left ventricular ejection fraction. GLS—global longitudinal strain.

**Table 2:** Characteristics of the patients.

Sex	
Male, n (%)	30 (54.5)
Female, n (%)	25 (45.5)
**Age,** years (median (minimum–maximum))	61 (18–74)
**Autologous transplantation, n (%)**	48 (87.3)
**Main disease**	
Multiple myeloma, n (%)	33 (68.8)
Mantle cell lymphoma, n (%)	4 (8.3)
Hodgkin’s lymphoma, n (%)	2 (4.2)
PCNS diffuse large B cell lymphoma, n (%)	5 (10.4)
Peripheral T cell lymphoma, n (%)	1 (2.1)
Ewing sarcoma, n (%)	2 (4.2)
NK-/T-cell lymphoma, n (%)	1 (2.1)
**Allogeneic transplantation, n (%)**	7 (12.7)
**Main disease**	
Acute myeloid leukemia, n (%)	5 (71.4)
Acute myelomonocytic leukemia, n (%)	1 (14.3)
MDS-EB, n (%)	1 (14.3)

PCNS—primary central nervous system, NK—natural killer, MDS-EB—myelodysplastic syndrome with excess blasts.

**Table 3:** Characteristics of the patients with and without CTRCD.

Characteristic	All Patients (n = 55)	Non-CTRCD (n = 40; 72.7%)	CTRCD (n = 15; 27.3%)	*p*
*Age* (years), median (Min–max)	61 (18–74)	61 (18–74)	61 (23–74)	0.502
*Sex*				
Male	30 (54.5)	21 (52.5)	9 (60.0)	0.764
Female	25 (45.5)	19 (47.5)	6 (40.0)	
*Disease*				
Multiple myeloma	33 (60.0)	27 (67.5)	6 (40.0)	0.121
Lymphoma	12 (23.6)	7 (17.5)	6 (40.0)	0.151
Leukemia and MDS-EB	7 (12.7)	4 (10.0)	3 (20.0)	0.376
Other diseases (Ewing sarcoma)	2 (3.6)	2 (5.0)	0 (0.0)	1.000
*Auto/allo*				
Allogeneic HSCT	7 (12.7)	4 (10.0)	3 (20.0)	0.376
Autologous HSCT	48 (87.3)	36 (90.0)	12 (80.0)	
*CVD risk factors*				
CAD	3 (5.5)	1 (2.5)	2 (13.3)	0.177
Arterial hypertension	21 (38.2)	15 (37.5)	6 (40.0)	1.000
Diabetes mellitus	4 (7.3)	4 (10.0)	0 (0.0)	0.565
Family history of CAD	8 (14.5)	6 (15.0)	2 (13.3)	1.000
Dyslipidemia	43 (78.2)	31 (77.5)	12 (80.0)	1.000
Previous smoking	5 (9.1)	4 (10.0)	1 (6.7)	1.000
*Medications*				
Beta-blockers	12 (21.8)	10 (25.0)	2 (13.3)	0.477
ACEis	10 (18.2)	7 (17.5)	3 (20.0)	1.000
ARBs	5 (9.1)	3 (7.5)	2 (13.3)	0.606
Statins	6 (10.9)	4 (10.0)	2 (13.3)	0.660
*Previous use of anthracyclines*	18 (32.7)	9 (22.5)	9 (60.0)	0.021
*Conditioning regimen*				
Melphalan	35 (63.6)	28 (70.0)	7 (46.7)	0.128
BEAM	7 (12.7)	2 (5.0)	5 (33.3)	0.013
Carmustine+TT	5 (9.1)	5 (12.5)	0 (0.0)	0.308
RIC	7 (12.7)	4 (10.0)	3 (20.0)	0.376
*TnI*, median (min–max)	0.02 (0.02–0.64)	0.02 (0.02–0.64)	0.02 (0.02–0.16)	0.958
*BNP*, median (min–max)	21.75 (4.00–118.70)	19.90 (4.00–118.70)	23.60 (9.30–56.20)	0.719

MDS-EB: myelodysplastic syndrome with excess blasts; HSCT: hematopoietic stem cell transplantation; CV: cardiovascular; CAD: coronary artery disease; ACEis: angiotensin converting enzyme inhibitors; ARBs: angiotensin receptor blockers; BEAM: carmustine, etoposide, cytarabine, melphalan; TT: thiotepa; RIC: reduced intensity conditioning.

**Table 4:** Univariate logistic regression analysis of factors possibly influencing the development of CTRCD.

Univariate Logistic Regression			
Covariate	OR	95% CI	*p*
Sex (male versus female)	1.357	0.407–4.529	0.619
Multiple myeloma (myeloma versus other disease)	0.321	0.094–1.095	0.069
Lymphoma (lymphoma versus other disease)	3.143	0.843–11.720	0.088
Allogeneic HSCT (allo versus auto)	2.250	0.439–11.522	0.330
Autologous HSCT (auto versus allo)	0.444	0.087–2.276	0.330
CAD (present versus absent)	6.000	0.502–71.731	0.157
Arterial hypertension (present versus absent)	1.111	0.330–3.746	0.865
Family history of CAD (yes versus no)	0.872	0.156–4.884	0.876
Dyslipidemia (present versus absent)	1.161	0.268–5.034	0.842
Previous smoking (yes versus no)	0.643	0.066–6.264	0.704
Beta-blockers (use versus non-use)	0.462	0.088–2.408	0.359
ACEis (use versus non-use)	1.179	0.262–5.310	0.831
ARBs (use versus non-use)	1.897	0.285–12.654	0.508
Statins (use versus non-use)	1.385	0.226–8.477	0.725
Previous use of anthracyclines (use versus non-use)	5.167	1.448–18.433	0.011
Melphalan used for conditioning (use versus non-use)	0.375	0.111–1.269	0.115
BEAM used for conditioning (use versus non-use)	9.500	1.599–56.426	0.013

HSCT: hematopoietic stem cell transplantation; CAD: coronary artery disease; ACEis: angiotensin converting enzyme inhibitors; ARBs: angiotensin receptor blockers; BEAM: carmustine, etoposide, cytarabine, melphalan.

**Table 5:** Multivariate logistic regression analysis of factors possibly influencing the development of CTRCD.

		Multivariate Model			Bootstrap Method	
Covariate		OR	95% CI	*p*	95% Bca CI	*p*
Risk factors	CAD	3.701	0.172–79.583	0.403	−20.156–38.064	0.085
	Arterial hypertension	0.568	0.120–2.692	0.476	−36.130–1.889	0.484
	Family history of CAD	0.355	0.035–3.642	0.384	−35.882–0.743	0.231
	Dyslipidaemia	3.893	0.476–31.860	0.205	−1.423–73.298	0.099
	Previous smoking	1.224	0.105–14.270	0.872	−20.161–2.551	0.545
Previous use of anthracyclines		3.913	0.712–21.501	0.117	−19.701–47.749	0.092
BEAM		6.654	0.660–67.061	0.108	−0.605–42.489	0.039

CAD: coronary artery disease; BEAM: carmustine, etoposide, cytarabine, melphalan; OR: odds ratio; CI: confidence interval; 95% BCa CI: Bias-corrected and accelerated confidence interval.

**Table 6:** Multivariate logistic regression analysis. Cardiovascular risk factors and conditioning regimen possibly influencing the development of CTRCD.

		Multivariate Model			Bootstrap Method	
Covariate		OR	95% CI	*p*	95% Bca CI	*p*
Risk factors	CAD	4.868	0.261–90.681	0.289	−20.403–24.326	0.062
	Arterial hypertension	0.614	0.133–2.841	0.533	−35.672–2.044	0.552
	Family history of CAD	0.398	0.043–3.688	0.418	−37.910–1.255	0.297
	Dyslipidaemia	2.729	0.351–21.220	0.337	−1.589–55.820	0.257
	Previous smoking	0.858	0.080–9.225	0.900	−20.385–1.907	0.549
BEAM		14.910	1.764–126.038	0.013	−0.469–41.661	0.006

CAD: coronary artery disease; BEAM: carmustine, etoposide, cytarabine, melphalan; OR: odds ratio; CI: confidence interval; 95% BCa CI: Bias-corrected and accelerated confidence interval.

**Table 7:** Multivariate logistic regression analysis. Cardiovascular risk factors and previous use of anthracyclines possibly influencing the development of CTRCD.

		Multivariate Model			Bootstrap Method	
Covariate		OR	95% CI	*p*	95% Bca CI	*p*
Risk factors	CAD	4.131	0.216–79.078	0.346	−19.808–22.766	0.075
	Arterial hypertension	0.595	0.133–2.662	0.497	−3.286–1.312	0.522
	Family history of CAD	0.597	0.078–4.561	0.619	−20.325–1.024	0.437
	Dyslipidaemia	2.643	0.419–16.692	0.301	−1.072–21.330	0.235
	Previous smoking	1.295	0.112–14.945	0.836	−20.105–2.645	0.508
Previous use of anthracyclines		6.996	1.530–31.997	0.012	−0.100–37.590	0.009

CAD: coronary artery disease; OR: odds ratio; CI: confidence interval; 95% BCa CI: Bias-corrected and accelerated confidence interval.

## Data Availability

The data presented in this study are available on request from the corresponding author.

## Supplementary Materials

The following supporting information can be downloaded at: https://www.mdpi.com/article/10.3390/cancers16223808/s1, Table S1: Characteristics of the patients with cancer therapy related dysfunction.

## References

[1] Zhao Y., He R., Oerther S., Zhou W., Vosough M., Hassan M. (2022). Cardiovascular Complications in Hematopoietic Stem Cell Transplanted Patients. J. Pers. Med..

[2] Ryan T.D., Hayek S., Rotz S. (2021). Review of Late CV Effects After Hematopoietic Stem Cell Transplantation. Am. Coll. Cardiol..

[3] Lawless S., Iacobelli S., Knelange N.S., Chevallier P., Blaise D., Milpied N., Foà R., Cornelissen J.J., Lioure B., Benjamin R. (2022). Comparison of autologous and allogeneic hematopoietic cell transplantation strategies in patients with primary plasma cell leukemia, with dynamic prediction modeling. Haematologica.

[4] Niederwieser D., Baldomero H., Bazuaye N., Bupp C., Chaudhri N., Corbacioglu S., Elhaddad A., Frutos C., Galeano S., Hamad N. (2022). One and a half million hematopoietic stem cell transplants: Continuous and differential improvement in worldwide access with the use of non-identical family donors. Haematologica.

[5] Ohmoto A., Fuji S. (2021). Cardiac complications associated with hematopoietic stem-cell transplantation. Bone Marrow Transpl..

[6] Blaes A., Konety S., Hurley P. (2016). Cardiovascular Complications of Hematopoietic Stem Cell Transplantation. Curr. Treat. Options Cardiovasc. Med..

[7] Rotz S.J., Ryan T.D., Hayek S.S. (2021). Cardiovascular Disease and its Management in Children and Adults Undergoing Hematopoietic Stem Cell Transplantation. J. Thromb. Thrombolysis.

[8] Jacob S.W., de la Torre J.C. (2009). Pharmacology of dimethyl sulfoxide in cardiac and CNS damage. Pharmacol. Rep..

[9] Lyon A.R., Lopez-Fernandez T., Couch L.S., Asteggiano R., Aznar M.C., Bergler-Klein J., Boriani G., Cardinale D., Cordoba R., Cosyns B. (2022). 2022 ESC Guidelines on cardio-oncology developed in collaboration with the European Hematology Association (EHA), the European Society for Therapeutic Radiology and Oncology (ESTRO) and the International Cardio-Oncology Society (IC-OS). Eur. Heart J..

[10] Williams B., Mancia G., Spiering W., Agabiti Rosei E., Azizi M., Burnier M., Clement D.L., Coca A., de Simone G., Dominiczak A. (2018). 2018 ESC/ESH Guidelines for the management of arterial hypertension: The Task Force for the management of arterial hypertension of the European Society of Cardiology and the European Society of Hypertension: The Task Force for the management of arterial hypertension of the European Society of Cardiology and the European Society of Hypertension. J. Hypertens..

[11] Lang R.M., Badano L.P., Mor-Avi V., Afilalo J., Armstrong A., Ernande L., Flachskampf F.A., Foster E., Goldstein S.A., Kuznetsova T. (2015). Recommendations for Cardiac Chamber Quantification by Echocardiography in Adults: An Update from the American Society of Echocardiography and the European Association of Cardiovascular Imaging. Eur. Heart J. Cardiovasc. Imaging.

[12] Moriyama S., Fukata M., Hieda M., Yokoyama T., Yoshimoto G., Kusaba H., Nakashima Y., Miyamoto T., Maruyama T., Akashi K. (2022). Early-onset cardiac dysfunction following allogeneic haematopoietic stem cell transplantation. Open Heart.

[13] Murbraech K., Smeland K.B., Holte H., Loge J.H., Lund M.B., Wethal T., Holte E., Rösner A., Dalen H., Kvaløy S. (2015). Heart failure and asymptomatic left ventricular systolic dysfunction in lymphoma survivors treated with autologous stem-cell transplantation: A national cross-sectional study. J. Clin. Oncol..

[14] Liu R., Sun F., Rampoldi A., Maxwell J.T., Wu R., Fischbach P. (2020). Melphalan induces cardiotoxicity through oxidative stress in cardiomyocytes derived from human induced pluripotent stem cells. Stem Cell Res. Ther..

[15] Thomas S.A. (2017). Chemotherapy Agents That Cause Cardiotoxicity. US Pharm..

[16] Sarzhevskiy V., Kolesnikova D., Melnichenko V., Vakhromeeva M. (2014). Cardiotoxicity of High-Dose Chemotherapy with Autologous Hematopoietic Stem Cells Transplantation in Patients with Malignant Lymphomas. what is Worse-Beam or Cbv?. Ann. Oncol..

[17] Bhagat A., Kleinerman E.S. (2020). Anthracycline-Induced Cardiotoxicity: Causes, Mechanisms, and Prevention. Adv. Exp. Med. Biol..

[18] Armenian S.H., Sun C.L., Shannon T., Mills G., Francisco L., Venkataraman K., Wong F.L., Forman S.J., Bhatia S. (2011). Incidence and Predictors of Congestive Heart Failure After Autologous Hematopoietic Cell Transplantation. Blood.

[19] Fujimaki K., Maruta A., Yoshida M., Sakai R., Tanabe J., Koharazawa H., Kodama F., Asahina S., Minamizawa M., Matsuzaki M. (2001). Severe Cardiac Toxicity in Hematological Stem Cell Transplantation: Predictive Value of Reduced Left Ventricular Ejection Fraction. Bone Marrow Transpl..

[20] Massey R.J., Diep P.P., Ruud E., Burman M.M., Kvaslerud A.B., Brinch L., Aakhus S., Gullestad L.L., Beitnes J.O. (2020). Left Ventricular Systolic Function in Long-Term Survivors of Allogeneic Hematopoietic Stem Cell Transplantation. JACC CardioOncol..

[21] Voß F., Nienhaus F., Pietrucha S., Ruckhäberle E., Fehm T., Melz T., Cramer M., Haberkorn S.M., Flögel U., Westenfeld R. (2024). Anthracycline Therapy Induces an Early Decline of Cardiac Contractility in Low-Risk Patients with Breast Cancer. Cardio Oncol..

[22] Di Lisi D., Madaudo C., Di Fazio L., Gulotta A., Triolo O.F., Galassi A.R., Incorvaia L., Russo A., Novo G. (2023). Higher Incidence of Cancer Therapy-Related Cardiac Dysfunction in the COVID-19 Era: A Single Cardio-Oncology Center Experience. J. Cardiovasc. Dev. Dis..

[23] Mauro C., Capone V., Cocchia R., Cademartiri F., Riccardi F., Arcopinto M., Alshahid M., Anwar K., Carafa M., Carbone A. (2023). Cardiovascular Side Effects of Anthracyclines and HER2 Inhibitors among Patients with Breast Cancer: A Multidisciplinary Stepwise Approach for Prevention, Early Detection, and Treatment. J. Clin. Med..

[24] Omland T., Heck S.L., Gulati G. (2022). The Role of Cardioprotection in Cancer Therapy Cardiotoxicity: JACC: CardioOncology State-of-the-Art Review. JACC CardioOncol..

[25] Linders A.N., Dias I.B., López Fernández T., Tocchetti C.G., Bomer N., Van der Meer P. (2024). A Review of the Pathophysiological Mechanisms of Doxorubicin-Induced Cardiotoxicity and Aging. npj Aging.

[26] Adão R., De Keulenaer G., Leite-Moreira A., Brás-Silva C. (2013). Cardiotoxicity Associated with Cancer Therapy: Pathophysiology and Prevention. Rev. Port. Cardiol..

[27] Raj S., Franco V.I., Lipshultz S.E. (2014). Anthracycline-Induced Cardiotoxicity: A Review of Pathophysiology, Diagnosis, and Treatment. Curr. Treat. Options Cardio Med..

[28] Henriksen P.A. (2018). Anthracycline cardiotoxicity: An update on mechanisms, monitoring and prevention. Heart.

[29] Fradley M.G. (2023). Heart Failure in Patients with Cancer Treated With Anthracyclines—Revisiting the Foundation of Cardio-Oncology. JAMA Netw. Open.

[30] Dempke W.C., Zielinski R., Winkler C., Silberman S., Reuther S., Priebe W. (2023). Anthracycline-Induced Cardiotoxicity—Are We About to Clear This Hurdle?. Eur. J. Cancer.

[31] Chaulin A.M. (2023). The Essential Strategies to Mitigate Cardiotoxicity Caused by Doxorubicin. Life.

[32] Tini G., Tocci G., Battistoni A., Sarocchi M., Pietrantoni C., Russo D., Musumeci B., Savoia C., Volpe M., Spallarossa P. (2023). Role of Arterial Hypertension and Hypertension-Mediated Organ Damage in Cardiotoxicity of Anticancer Therapies. Curr. Heart Fail Rep..

[33] Vaitiekus D., Muckiene G., Vaitiekiene A., Maciuliene D., Vaiciuliene D., Ambrazeviciute G., Sereikaite L., Verikas D., Jurkevičius R., Juozaityte E. (2020). Impact of arterial hypertension on Doxorubicin-Based Chemotherapy-Induced subclinical cardiac damage in breast cancer patients. Cardiovasc. Toxicol..

