# Supplementary material for: Evaluation of Subclinical Cancer Therapy-Related Cardiac Dysfunction in Patients Undergoing Hematopoietic Stem Cell Transplantation: An Echocardiography Study
Source: Cancers (Basel). 2024 Nov 12;16(22):3808. doi: 10.3390/cancers16223808 (PMC11592334; doi:10.3390/cancers16223808)
Supplement: Supplementary file 1 [file cancers-16-03808-s001.zip › cancers-3249473-supplementary.pdf]

Table S1: Characteristics of the patients with cancer therapy related dysfunction

Patient	Sex	Age	Transplantation type	Main disease	CAD	Arterial hypertension	Diabetes mellitus	Family history of CAD	Dyslipidemia	Previous smoking	Previous use of anthracyclines	Conditioning regimen	LVEF, % (1)	LVEF, % (2)	GLS, % (1)	GLS, % (2)
1	male	38	Autologous	NK-/T-cell lymphoma	No	No	No	No	No	No	Yes	BEAM	48.6	47.6	-14.3	-12.0
2	male	52	Autologous	Multiple myeloma	No	No	No	No	Yes	No	No	Melphalan	63.3	49.4	-9.1	-8.0
3	female	63	Autologous	Mantle cell lymphoma	No	Yes	No	No	Yes	No	Yes	BEAM	62.0	53.0	-16.3	-13.0
4	male	23	Autologous	Hodgkin's lymphoma	No	No	No	No	No	No	Yes	BEAM	62.1	43.0	-15.1	-10.3
5	male	63	Autologous	Multiple myeloma	No	Yes	No	No	Yes	No	No	Melphalan	58.1	54.0	-22.9	-18.0
6	female	54	Allogeneic	Acute myeloid leukaemia	No	Yes	No	No	No	No	Yes	RIC	59.9	56.2	-21.6	-12.0
7	female	45	Autologous	Multiple myeloma	No	No	No	No	Yes	No	No	Melphalan	63.5	45.4	-16.0	-17.5
8	male	61	Autologous	Mantle cell lymphoma	No	No	No	Yes	Yes	No	Yes	BEAM	65.5	60.0	-18.6	-15.8
9	female	55	Autologous	Mantle cell lymphoma	No	No	No	No	Yes	No	Yes	Melphalan	49.6	46.1	-19.3	-15.0
10	male	65	Allogeneic	Acute myeloid leukaemia	Yes	Yes	No	No	Yes	No	Yes	RIC	51.6	50.0	-17.8	-14.7
11	female	49	Autologous	Multiple myeloma	No	No	No	No	Yes	No	No	Melphalan	62.3	55.1	-22.3	-18.7
12	male	61	Autologous	Multiple myeloma	No	No	No	No	Yes	Yes	No	Melphalan	60.0	62.7	-19.4	-9.6
13	female	67	Autologous	Multiple myeloma	No	Yes	No	No	Yes	No	No	Melphalan	62.6	55.0	-23.3	-16.5
14	male	74	Autologous	Peripheral T cell lymphoma	Yes	Yes	No	No	Yes	No	Yes	BEAM	48.3	45.0	-14.2	-10.8
15	male	69	Allogeneic	Acute myeloid leukaemia	No	No	No	Yes	Yes	No	Yes	RIC	52.3	62.8	-15.5	-12.6

CAD: coronary artery disease; BEAM: carmustine, etoposide, cytarabine, melphalan; RIC: reduced intensity conditioning; LVEF: left ventricular ejection fraction; GLS: global longitudinal strain; (1): baseline; (2): control.
